# LncRNA DANCR Enhances Angiogenesis to Promote Melanoma Progression Via Sponging miR-5194

**DOI:** 10.7150/jca.81723

**Published:** 2023-05-05

**Authors:** Jing Jia, Xinxi Zhu, Kaihua Xue, Yuanmei Huang, Menglu Wu, Yanan Yang, Wenbo Liu, Hongke Zhang, Lin He, Hong Sun

**Affiliations:** 1 Department of Plastic, Cosmetic and Maxilofacial, The First Affiliated Hospital of Xi'an Jiaotong University, Xi'an, Shaanxi, China;; 2 Department of Urology, The First Affiliated Hospital of Xi'an Jiaotong University, Xi'an, Shaanxi, China;; 3 Honghui Hospital, Xi'an, Shaanxi, China;; 4 Department of Medical Oncology, The First Affiliated Hospital of Xi'an Jiaotong University, Xi'an, Shaanxi, China.

**Keywords:** LncRNA DANCR, angiogenesis, melanoma, miR-5194, VEGFB

## Abstract

**Background and aim:** As an oncogenic long noncoding RNA, differentiation antagonizing non-protein coding RNA (DANCR) was identified in many kinds of cancers. However, the specific function of DANCR in melanoma remains unclear. Here, we aimed to clarify the role of DANCR played in melanoma progression and the underlying mechanisms.

**Methods:** TCGA data base and patients' tissue samples were used to analyzed the function of DANCR in melanoma progression. Transwell assay was used to detect cell migration and tube formation assay was employed to assess the ability of angiogenesis. Western blot, qRT-PCR, ELISA and IHC assay were used to examine VEGFB expression and secrection. Luciferase assay verified the binding of DANCR and miRNA.

**Results:** We found that the expression of DANCR was positively related to poor clinical prognosis of melanoma. DANCR knockdown suppressed melanoma progression with a more significant suppression *in vivo* compared with it *in vitro*. Further detection showed that beyond promoting proliferation, DANCR also enhanced angiogenesis *via* upregulating VEGFB. Mechanistic analysis revealed that DANCR upregulating VEGFB through sponging miR-5194, which negatively regulated VEGFB expression and secretion.

**Conclusion:** We demonstrated a novel oncogenic role DANCR played in melanoma and suggested a new avenue for melanoma therapy by targeting the DANCR/miR-5194/VEGFB signaling.

## Introduction

As the 7th most frequent malignant cancer in females and the 5th most frequent malignant cancer in males worldwide, melanoma, originating from skin melanocytes, is one of the most life-threatening tumors 1. More terribly, melanoma metastasis and progression usually happen rapidly and remarkable increase of melanoma incidence was found in recent years 2,3. Hence, creating innovative therapeutic strategies for melanoma treatment is necessary. Immune-associated approaches and chemotherapy have been developed, which showed advantages in improving melanoma management. However, therapeutic resistance and drug toxicity bring challenges for melanoma patients 4,5. Accordingly, elucidating the underlying mechanisms involved in melanoma progression and identifying new therapeutic targets is extremely urgent.

Increasing evidences underlined the important role of lncRNAs in modulating biological activities of melanoma cells, including but not limited to cell migration 6, cell proliferation 7 and cell autophagy 8. Although expression of lncRNAs have a higher cell-type specificity and lower expression level compared with protein coding mRNAs 9, dysfunction or dysregulation of lncRNAs have been proofed to relate to initiation, progression and relapse of tumors 10. A plenty of studies have revealed the regulator role lncRNAs played in melanoma occurrence and development. For instance, lncRNA positioned on chromosome 1 was amplified to enrich enhancer of zeste homolog 2 at the promoter of p21, which sequentially suppressed p21 transcription and enhanced melanoma cell proliferation 11. Differentiation antagonizing non-protein coding RNA (DANCR) was initially found as a pair lncRNA with a length of 855 base, that was downregulated during the progress of differentiation 12. Moreover, expression of DANCR was dysregulated in several cancers, such as prostate cancer 13,14, hepatoma 15, glioma 16 and lung cancer 17. However, the function of DANCR in melanoma development is rarely studies.

Emerging evidences exposed diverse molecular mechanisms of DANCR regulating target gene expression. Functioned as ceRNA, DANCR sponged with miR-335-5p to regulate ROCK1 expression, as well as invasion, migration and proliferation of cervical cancer cells 18. Besides, DANCR sponges miR-135a to regulate prostate cancer sensitivity to paclitaxel 14. Furtherance, DANCR also functioned as ceRNA to promote proliferation of hepatocellular carcinoma through suppressing cell apoptosis and cell cycling 19. Moreover, DANCR could interact with epigenetic gene silencer, EZH2, to reduce the expression of TIMP2 and TIMP3 and promote prostate cancer invasion and migration 13.

Regarding these and the important role of DANCR in several tumorigenesis, in present study, we explored the function of DANCR in regulating melanoma progression as well as the underlying molecular mechanism.

## Methods and materials

### Cell lines, RNA interference and conditioned medium (CM) collection

Human melanoma cell clones, A375 and SK-MEL-28 cells, were bought from American Type Culture Collection (Manassas, VA, US), which were raised with DMEM supplemented with fetal bovine serum (10%) with a humidified atmosphere keeping the temperature of 37°C and CO_2_ concentration of 5%. DANCR stably knockdown melanoma cells and the corresponding negative control cells were created using short hairpin (sh) RNA specifically targeting DANCR or non-specific control shRNA contained in the replication defective Lentivirus, and the cells were named as shDANCR or shNC cells respectively. Lipofectamine 2000 reagent (Invitrogen, CA, US) was employed to transfected small interfering (si) RNA to knocking down DANCR or overexpressing miR-5194. miR-5194 inhibitor, purchased from RiboBio (Guangzhou, China), was transfected into cells according to manufactures protocel. Differently treated cells were seeded into 6 cm culture dish with 5×10^5^ cell per dish. 24 h later, cells were washed with serum free medium (SFM) triply and cultured with SFM (5ml) for another 24 h. Then the medium was collected and centrifuged to collect the supernatants. The collected CM was used immediately or stored at -80°C until usage.

### Melanoma tissues collection and analysis

With approval and supervision from the institutional review board and informed consents from all patients, we collected forty melanoma tissues from patients accepted treatment in the First Affiliated Hospital of Xi'an Jiaotong University. All patients participated in this study did not treat with any preoperative therapy, including chemotherapy, immunotherapy and radiotherapy, with their diagnoses determined according to the histopathological evidences.Fresh tissues were carefully washed with sterile PBS (phosphate buffered saline) and stored at -80°C immediately after surgery for subsequent RNA extraction. For IHC assay (immunohistochemical assay), the tissues were fixed with paraformaldehyde, embedded with paraffin and cut into sections with the thickness of 5 μm before staining with primary antibody against CD31 or VEGFB using the DAKO Autostainer Plus system. Pathologist double-blindly viewed and analyzed the sections under high power (× 400) field.

### Bioinformatics

Melanoma microarray data about RNA sequencing of melanoma samples was downloaded from TCGA Data portal. The data were normalized to Z score before statistical analysis.

### Animal experiments

A total of 12 BALB/c-nude mice, half male and half female, were allocated to two groups and randomly digitized. When the mice were 5-week-old, shDANCR or shNC A375 cells (2 × 10^6^) were subcutaneously injected into the right ventral of every mouse. All mice tested their body weight every 5 days. After 4 weeks, the mice were sacrificed and the tumors were surgically harvested previous to immediate storage at -80°C for extraction of total RNA and fixed with paraformaldehyde for IHC assay. All of the animal experiments were accomplished following the guide of the institutional review board of the first affiliated hospital of the Xi'an Jiaotong University.

### Real-time quantitative- (qRT-) PCR analysis

Trizol reagent (Life Technologies, Rockville, MD, USA) was used to extract total RNA before reverse transcription into cDNA with PrimeScriptTM RT reagent kit purchased from Takara (Dalian, China). Next, CFX96 real-time PCR system (Bio-Rad, Hercules, CA) was used to examine the expression of interested targets with SYBR Green PCR Master Mix (Takara) and specific primers. GAPDH was employed as the internal control for normalization. Sequences of the primers for qRT-PCR are as following:

DANCR: forward-5′-GCCACTATGTAGAGGGTTTC-3′ and reverse-5′-ACCTGCGCTAAGAACTGAGG-3′; VEGFB: forward-5'-ACCCCCAACCCTGATAAAAG-3' and reverse-5'-TCCTCATTTCCTCCATCTGC-3'; GAPDH: forward-5′-TCAAGGCTGAGAACGGGAAG-3′ and reverse-5′-TCGCCCCACTTGATTTTGGA-3′.

The primer of miR‐5194 was purchased from RiboBio and used according to the protocol of the manufacture.

### Western blot assay

Cold PBS washed cells two times previous to lysis with protease inhibitor added RIPA buffer containing Tris (50 mM, pH 8.0), NaCl (150 mM, 0.1% SDS), NP-40 (1%) and sodium deoxycholate (0.5%). Protein, 30 μg per sample, was separated with SDS-PAGE gel (10%) before blotting onto the nitrocellulose membranes. The membrane was incubated at room temperature with skim milk (5%) and washed with TBST washing buffer, before incubation with primary antibody against VEGFA (ab46154, Abcam) overnight at 4°C. After washing with TBST washing buffer, the membrane was incubated with horseradish peroxidase-conjugated secondary antibodies for 1 h at room temperature and visualized with Molecular Imagery ChemiDoe XRS System from Bio-Rad Laboratories.

### Distribution of cell cycle analysis

Cold PBS washed cells were trypsinized, resuspended in cold ethanol (70%) and stored at -20°C for more than 24 h. Then the cells were centrifuged to remove the ethanol and washed with cold PBS for two times before resuspension with PBS containing RNAse A (0.5 μg/ml) and propidium iodide (50 μg/ml). The resuspended cells were stored without light at room temperature for half an hour previous to subject to flow cytometry analysis.

### MTT assay

Differently treated cells (2×10^3^) were seeded into every well of the 96-well plate and cultured for various times. Then cells were washed sterile PBS and cultured with MTT solution (0.5 mg/ml). Four hours later, medium was carefully moved and 150 μl DMSO was added to each well. After the crystals were fully dissolved, the plate was subjected to the microplate automatic reader with a wavelength for 490 nm to detect the absorbance (Bio-Tek Instruments Inc., Winooski, VT, USA).

### Colony formation assay

Melanoma cells with or without DANCR knockdown were added into 6-well plate with 1000 cells/well. About 2 weeks later, the cells were washed with sterile PBS, fixed with paraformaldehyde (4%) and stained with crystal violet solution (0.1%). Then the number of visible cell colonies in each well were examined.

### ELISA assay

Concentration of secreted VEGFB in differently treated cells were detected with Human VEGFB ELISA kit (RayBiotech Inc, Norcross, GA, USA) using CM previously collected. To modify the concentration of VEGF in CMs, we used recombinant human VEGFB_167_ purchased from R&D Systems Inc. (Minneapolis, MN, USA) to add to the CMs.

### HUVEC migration analysis

Transwell migration system was used with 8-μm-pore inserts as the upper chamber (Millipore, Bedford, MA, USA). SFM suspending HUVECs with a concentration of 3×10^5^ cell/ml were added to the upper chamber (300 μl). Differently treated CMs (1 ml) or differently treated melanoma cells (0.5×10^5^) were added to the lower chamber as the chemoattractant. 16 hours later, cells in the upper chamber were fixed with paraformaldehyde (4%) and stained with crystal violet (0.1%) with carefully protecting cells in the lower surface of the chamber. After wiping the upper surface of the inserts with cotton swab, cells migrated to the lower surface of the inserts were counted.

### Tube formation assay

SFM or CM diluted SFM (1:1) suspended HUVECs (1×10^5^ per well) were added into the 24-well plate coated with matrigel. About 5 hours later, plate was taken out from the cell incubator to take photographs. Two branching points perfectly continuous tube was deemed as a tube.

### Dual luciferase assay

The miR-135a binding region of DANCR with or without mutation were amplified and inserted into the pGL3-basic plasmids by GeneCopoeia (Guangzhou, China), which were named as pGL3-DANCR and pGL3-mt. The constructed plasmids, presence or absence of miR-5194, were used to transfect cells with X-tremeGENE HP DNA transfection reagent purchased from Roche (Mannheim, Germany). Dual Luciferase Assay kit purchased from Promega (Madison, WI, USA) was used to perform the luciferase assay according to the manufacture's instruction. For each data point, cells seeded in 3 independent wells were detected.

### Statistical analysis

Differences between 2 groups were analyzed using student's t-test with GraphPad Prism (version 6.0, San Diego, CA, USA). Pearson's correlation and linear regression analysis were also performed with GraphPad. The *p* value less than 0.05 was identified as statistically significant.

## Results

### High DANCR expression was positively related to melanoma progression

**Figure 1:**
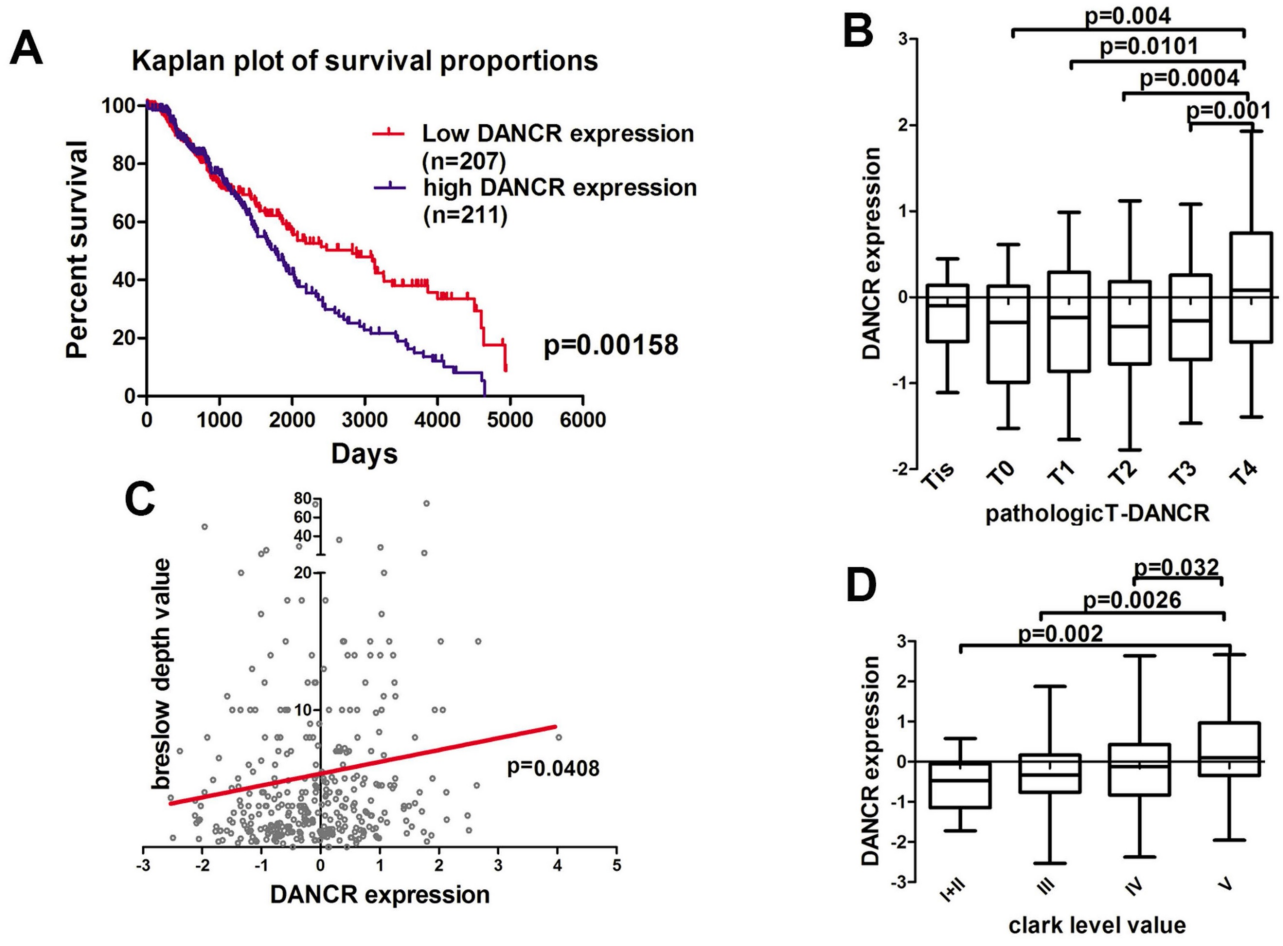
** Highly expressed DANCR correlated to poor clinical prognosis in melanoma patients. (A)** Kaplan-Meier analysis of the survival time of melanoma patients with high DANCR expression (n = 211) and low DANCR expression (n = 207) levels: the p-value is depicted. Data from the TCGA database. **(B)** Relative expression of DANCR in patients with different pathological T stages. T4: n = 141; T3: n = 70; T2: n = 69; T1: n = 36; T0: n = 22; Tis: n = 8. Data from the TCGA database. **(C)** Correlation of DANCR expression and breslow depth value: the p-value is depicted. Data from the TCGA database. **(D)** Relative expression of DANCR in patients with different clark levels. Clark level I and II: n = 23; Clark level III: n = 75; Clark level IV: n = 151; Clark level V: n = 49. Data from the TCGA database.

First of all, we analyzed the microarray data profiling of human melanoma tissues from the TCGA database to identify the DANCR expression in melanoma patients. According to the TCGA database, high-DANCR-expression group in melanoma had poorer overall survival comparing to low-DANCR-expression group (Figure 1A). Further analysis showed that high DANCR expression was associated with higher pathological T stage in melanoma (Figure 1B). Moreover, positive correlation was found between DANCR expression and Breslow depth level (Figure 1C). Besides, melanoma with higher Clark level value rendered higher DANCR expression (Figure 1D). Thus, clinical evidences suggested that DANCR played a tumor-promoting role in melanoma progression and closely associated with the poor clinical prognosis in melanoma patients.

### DANCR knockdown repressed proliferation of melanoma cells *in vivo* and *in vitro*

**Figure 2:**
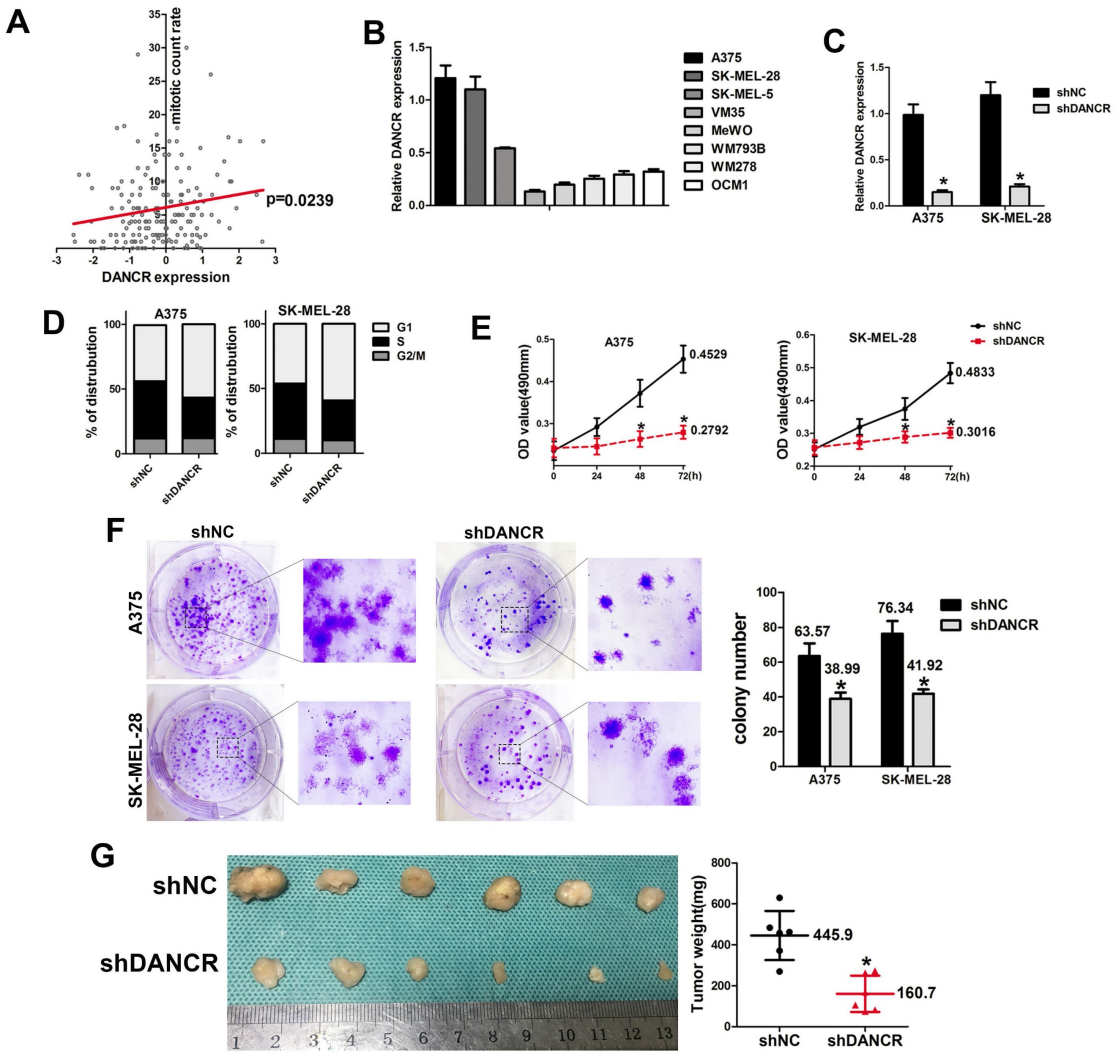
** DANCR knockdown reduced proliferation of melanoma cells *in vivo* and *in vitro*. A.** Correlation of DANCR expression and mitotic count rate: the p-value is depicted. Data from the TCGA database. **B.** Expression of DANCR in different melanoma cell lines. **C.** Lentivirus carrying shRNA targeting DANCR or the corresponding negative control shRNA were used to knock DANCR down in A375 and SK-MEL-28 cells, named shDANCR and shNC respectively. Expression of DANCR were examined by qRT-PCR assay. **D.** Distribution of cell cycling in A375 and SK-MEL-28 cells with or without DANCR knockdown. **E.** Cell viability of these cell clones was detected using MTT assay; Lower panel: colony number in different groups. **F.** Colony formation assay were performed in shDANCR and shNC cells. **G.** Subcutaneous xenograft tumor model using shDANCR-A375 cells or shNC-A375 cells was employed to investigate cell proliferation *in vivo*; Right panel: tumor weight of tumors in different groups. *p < 0.05.

Data from TCGA database also revealed that higher level of DANCR expression was accompanied with higher rate of mitotic cells (Figure 2A), which indicated that DANCR might accelerate cell proliferation in melanoma cells. For further exploration, we employed qRT-PCR assay to clarify the expression of DANCR in different melanoma cell lines and found highest expression level of DANCR in A375 and SK-MEL-28 cells comparing to other cell lines (Figure 2B). Thus, we knocked DANCR down in A375 and SK-MEL-28 cells with lentivirus harboring shRNA targeting DANCR (Figure 2C). Then we performed flow cytometry assay and investigated diminished cell proliferation induced by DANCR knockdown with more cells stayed at G1 stage (Figure 2D). Further detection with MTT assay also showed reduced cell viability in A375 and SK-MEL-28 cells with downregulated DANCR (Figure 2E). Consistently, colony formation assay further confirmed suppressed cell proliferation in A375 and SK-MEL-28 cells with downregulated DANCR (Figure 2F).

To verify the results *in vivo*, we used the subcutaneous xenograft tumor model with shDANCR-A375 and shNC-A375 cells injected into 4-week-old nude mice. After 4 weeks raising, no significant difference was found in body weight between two groups of mice (SFigure 1). All the mice were sacrificed and the tumors were harvested, which showed significantly reduced tumor weight and tumor volume in tumors constituted by shDANCR-A375 cells (Figure 2G).

Altogether, results in figure 1 indicated that downregulated DANCR could repress proliferation of melanoma cells. However, further analysis of cellular suppression showed about 40% decline of cell proliferation *in vitro* (Figure 2E and 2F), while a decline of 69.3% was found in tumor weight (Figure 2G). This information warns us that beyond proliferation DANCR also promoted melanoma progression through other means.

### DANCR concerned in the interaction between melanoma cell and HUVEC

**Figure 3:**
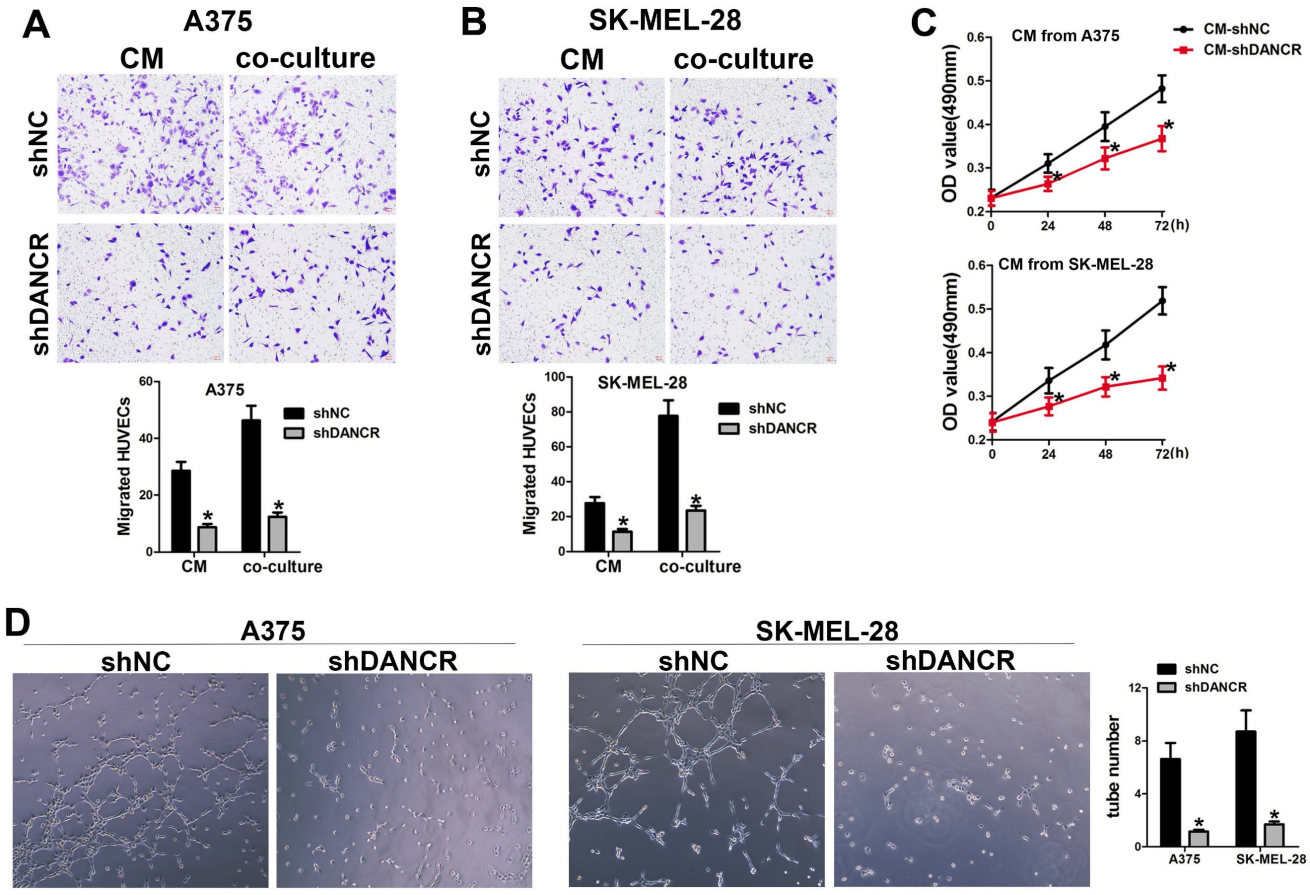
** DANCR concerns melanoma cell promoting angiogenesis. A** and **B**. HUVECs were seeded in the upper chamber of the Transwell system, with conditioned medium (CM) from A375 and SK-MEL-28 cells with or without DANCR knockdown in the lower chamber. Recruitment of HUVECs was detected; Right panel: quantitative analysis of recruited HUVECs. **C**. CM from DANCR knocking down melanoma cells treated HUVECs for different times and MTT assay was performed to examine cell viability of HUVECs. **D**. Tube formation ability of HUVECs with treatment of CM from differently treated A375 and SK-MEL-28 cells; Right panel: quantitative analysis of tube number. *p < 0.05.

In the subcutaneous xenograft tumor model, we found that tumors comprising of shNC-A375 cell were red, while tumors comprising of shDANCR-A375 cell were pale with less blood vessels could be seen on the surface. In according with the important role of angiogenesis in neoplasia, we hypothesized the function of DANCR in regulating angiogenesis in melanoma microenvironment. Hence, we collected conditioned medium (CM) from A375 and SK-MEL-28 cells with or without DANCR knockdown, which were put into the lower chamber of a transwell system with HUVECs in the upper inserts. Results in Figure 3A and 3B suggested reduced HUVEC migration with CM from DANCR knockdown melanoma cells. Furtherance, Figure 3A and 3B also showed that melanoma cells with DANCR knockdown seeded in the lower chamber recruited less HUVECs compared with melanoma cells absence of DANCR knockdown. Further analysis using MTT assay indicated weakened HUVEC cell viability with treatment of CM from shDANCR-A375 and shDANCR -SK-MEL-28 cells (Figure 3C). Moreover, CM from melanoma cells with DANCR knockdown diminished the ability of tube formation in HUVECs (Figure 3D).

Taken together, results in figure 3 showed that DANCR knockdown might reshape the activity of paracrine in melanoma cells and repress the interaction of melanoma cells with HUVECs.

### Knockdown DANCR downregulated VEGFB in melanoma cells

**Figure 4:**
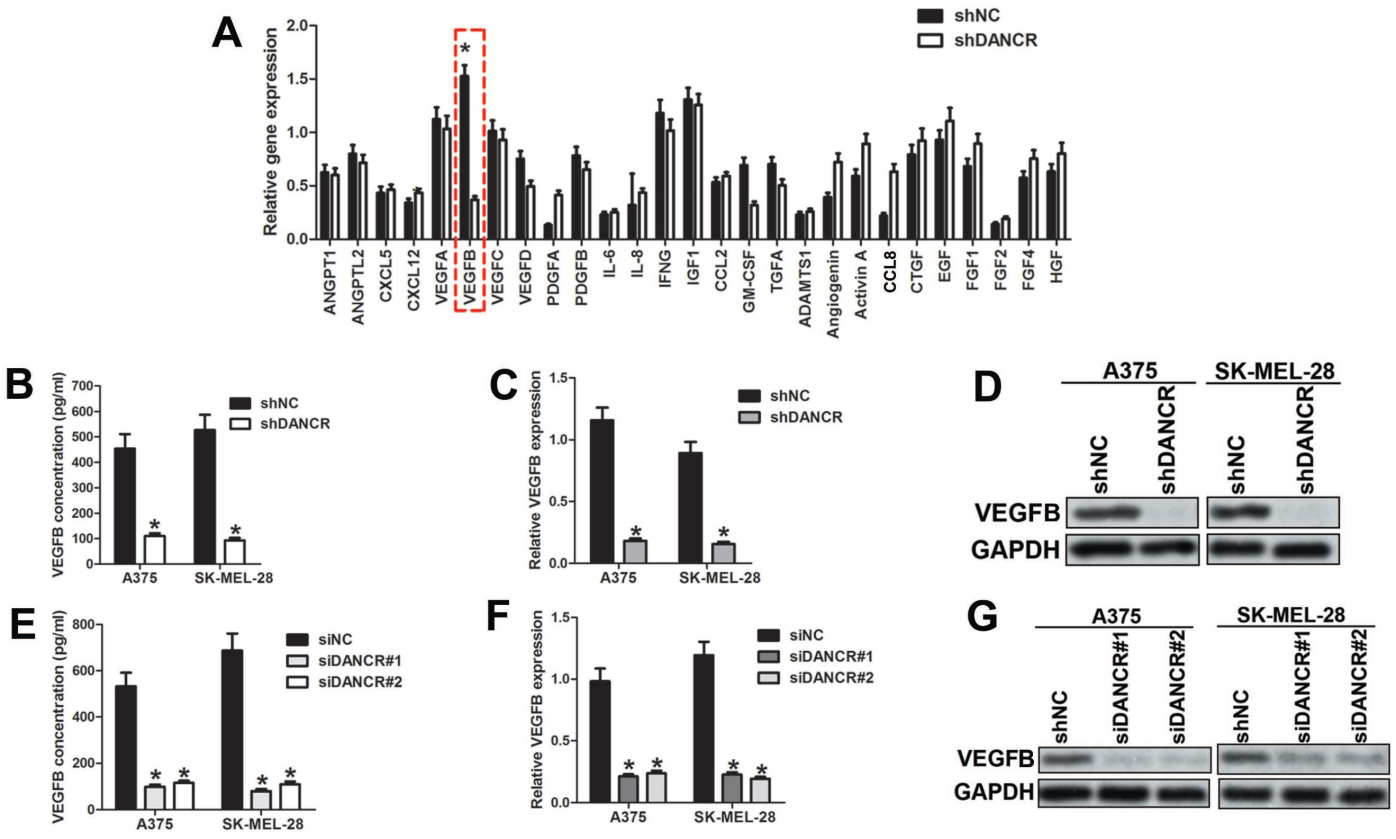
** DANCR knockdown suppresses expression of VEGFB. A.** Expression of angiogenic cytokines between A375 cells with or without DANCR knockdown were detected with qRT-PCR assay. **B.** CM were collected from A375 cells and SK-MEL-28 cells with or without DANCR knockdown previous to examination of VEGFB concentration using ELISA assay. **C** and **D.** Expression of VEGFB in mRNA level and protein level in above cells. **E.** DANCR was downregulated in A375 and SK-MEL-28 cells with siRNA and the secreted VEGFB in the medium was detected with ELISA assay. **F** and **G.** mRNA level and protein level of VEGFB in A375 and SK-MEL-28 cells with or without DANCR knockdown by siRNA. *p < 0.05.

To clarify the potential angiogenic factors involved in DANCR modulating interaction of melanoma cell and HUVECs, we implemented qRT-PCR assay to profile the different expressed angiogenic factors in A375 cells with or without DANCR knockdown. As shown in Figure 4A, VEGFB was dramatically downregulated in DANCR knockdown A375 cells among the 27 candidates. Considering the predominant role of VEGFB in modulating angiogenesis 20, we focused on VEGFB. Similarly, reduced VEGFB concentration was found in the medium of DANCR downregulated A375 and SK-MEL-28 cells (Figure 4B). Moreover, expression of VEGFB was also decreased in melanoma cells with DANCR knockdown detected by both qRT-PCR assay and Western Blot assay (Figure 4C and 4D). For further confirmation, we knocked DANCR down with siRNA, which suggested reduced VEGFB secretion and lessened VEGFB expression in both mRNA level and protein level (Figure 4E, 4F and 4G). Taken together, results in figure 4 showed that DANCR knockdown decreased VEGFB expression and secretion in melanoma cells.

### VEGFB played important role in melanoma cell promoting angiogenesis

**Figure 5:**
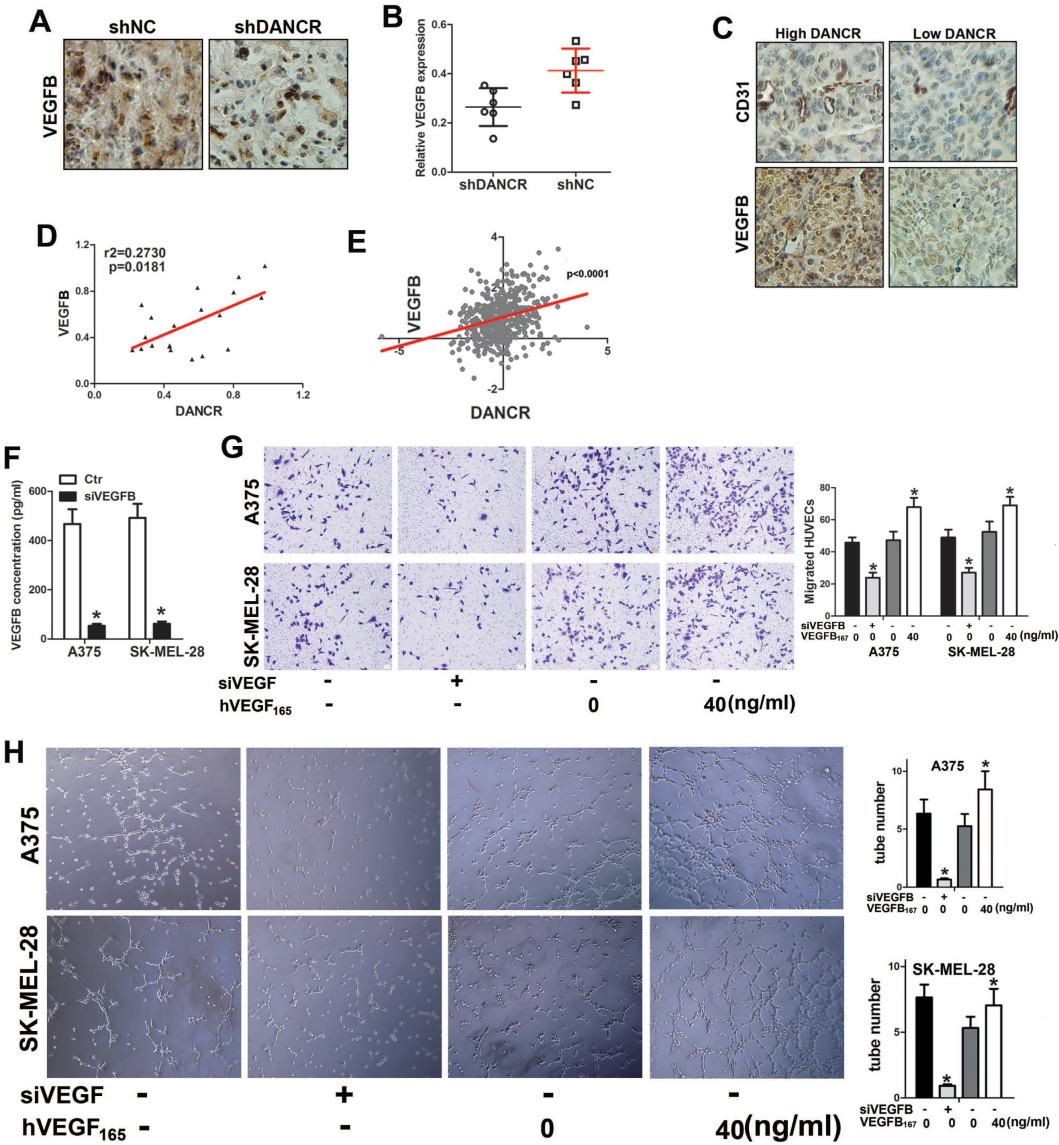
** VEGFB is essential for DANCR mediated melanoma cell promoting angiogenesis. A.** Expression of VEGFB in subcutaneous xenograft tumors comprising of A375 cells with or without DANCR knockdown. **B.** Quantitative analysis of VEGFB expression in different xenograft tumors. **C.** IHC assay with anti-CD31 and anti-VEGFB was performed in human melanoma tissues. Expression of CD31 and VEGFB in patients' melanoma tissues with lower and higher DANCR level. **D.** Linear regression analysis of DANCR expression and VEGFB expression. **E.** Correlation of DANCR expression and VEGFB expression: the p-value is depicted. Data from the TCGA database.** F.** Concentration of secreted VEGFB in melanoma cells with or without siRNA specifically targeting VEGFB. **G.** Melanoma cells without DANCR knockdown were treated with siRNA to knock VEGFB down. Melanoma cells with DANCR knockdown were treated with recombinant human VEGFB (VEGFB_167_). Analysis of HUVECs recruitment with CM from differently treated melanoma cells put into the lower chamber; Right panel: quantitative data of migrated cells. **H.** Tube formation assay of HUVECs with treatment of CM from above melanoma cells; Right panel: quantitative data of formed tubes. *p < 0.05.

To verify the function of VEGFB in modulating DANCR-involved angiogenesis in melanoma, we investigated VEGFB expression in the subcutaneous xenograft tumors. Unsurprisingly, suppressed VEGFB expression was found in DANCR knockdown subcutaneous tumors (Figure 5A and 5B). Consistently, weakened VEGFB expression was detected in human melanoma tissues with lower DANCR expression (Figure 5C and SFigure 2), together with sparse vessel density (Figure 5C). Furtherance, both our collected melanoma tissues and microarray data from TCGA data base showed positive correlation between DANCR and VEGFB in human melanoma tissues (Figure 5D and 5E).

For further verification, we knocked VEGFB down in shNC-melanoma cells to decrease the secreted VEGFB in the CM (Figure 5F) and added human recombinant VEGFB_167_ into the CM of shDANCR-melanoma cells. We found impaired HUVECs recruitment with CM from VEGFB downregulated shNC-melanoma cells. Consistently, VEGFB_167_ rescued the weakened HUVECs migration induced by shDANCR-melanoma cells (Figure 5G). Coincidentally, inhibition of VEGFB diminished the tube formation induced by shNC-melanoma cells and VEGFB_167_ reversed the reduction of tube formation caused by shDANCR-melanoma cells (Figure 5H).

Altogether, results from Figure 5 indicated that VEGFB played key role in DANCR modulating angiogenesis of melanoma.

### DANCR directly binds and sponges miR-5194 in melanoma cells

**Figure 6:**
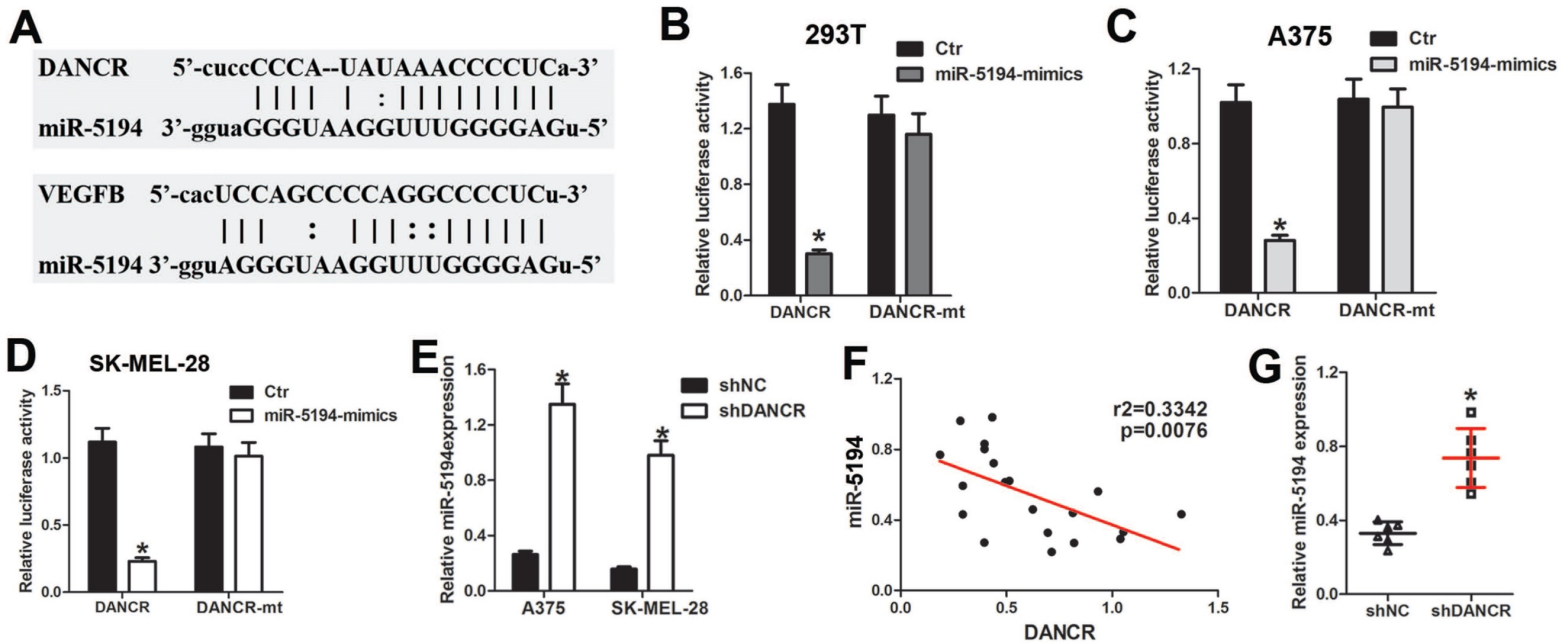
** DANCR interact with miR-5194 directly in melanoma cells. A.** Schematic diagram of the binding site of between miR-5194 and DANCR and VEGFB**. B.** DANCR-luciferase cells and DANCR-mutant-luciferase cells was transfected with miR-Ctr or miR-5194 before determination of luciferase activity in 293T cells. **C** and **D.** A375 and SK-MEL-28 cells were transfected with DANCR-luciferase plasmid or DANCR-mutant-luciferase plasmid together with miR-Ctr or miR-5194 previous to the analysis of luciferase activity. **E.** Expression of miR-5194 in A375 and SK-MEL-28 cells with transfection of shNC or shDANCR. **F.** Correlation of DANCR and miR5194 in human melanoma tissues. **G.** Expression of miR-5194 in xenograft tumors constituted by shDANCR-A375 cells or shNC-A375 cells. *p < 0.05.

As long noncoding RNA usually interact with miRNA to modulate its function of binding to target genes. We used Target Scan (http://www.targetscan.org/) and starBase (https://starbase.sysu.edu.cn/starbase2/index.php) to analyze the bioinformation of the potential target of DANCR, which indicated that miR-5194 could bind to both DANCR and VEGFB (Figure 6A). For further demonstration, DNA fragment of DANCR was cloned and inserted into the pGL3-basic luciferase reporter plasmid. Results of luciferase assay in 293T cells suggested that miR-5194 repressed the luciferase activity induced by DANCR translation, without affection on the mutant-DANCR (DANCR-mt) translation induced luciferase activity (Figure 6B). Further verification of the directly binding between DANCR and miR-5194 was demonstrated with luciferase assay performed in A375 and SK-MEL-28 cells (Figure 6C and 6D). Moreover, upregulated expression of miR-5194 was found in melanoma cells with DANCR knockdown (Figure 6E). Besides, elevated miR-5194 expression was shown in the xenograft tumors originating from shDANCR-A375 cells (Figure 6F). Consistently, expression of DANCR was negatively related to miR-5194 expression in human melanoma tissues (Figure 6G). Altogether, results in figure 6 demonstrated that DANCR could bind and sponge miR-5194 directly.

### miR-5194 plays key role in DANCR regulating VEGFB expression

**Figure 7:**
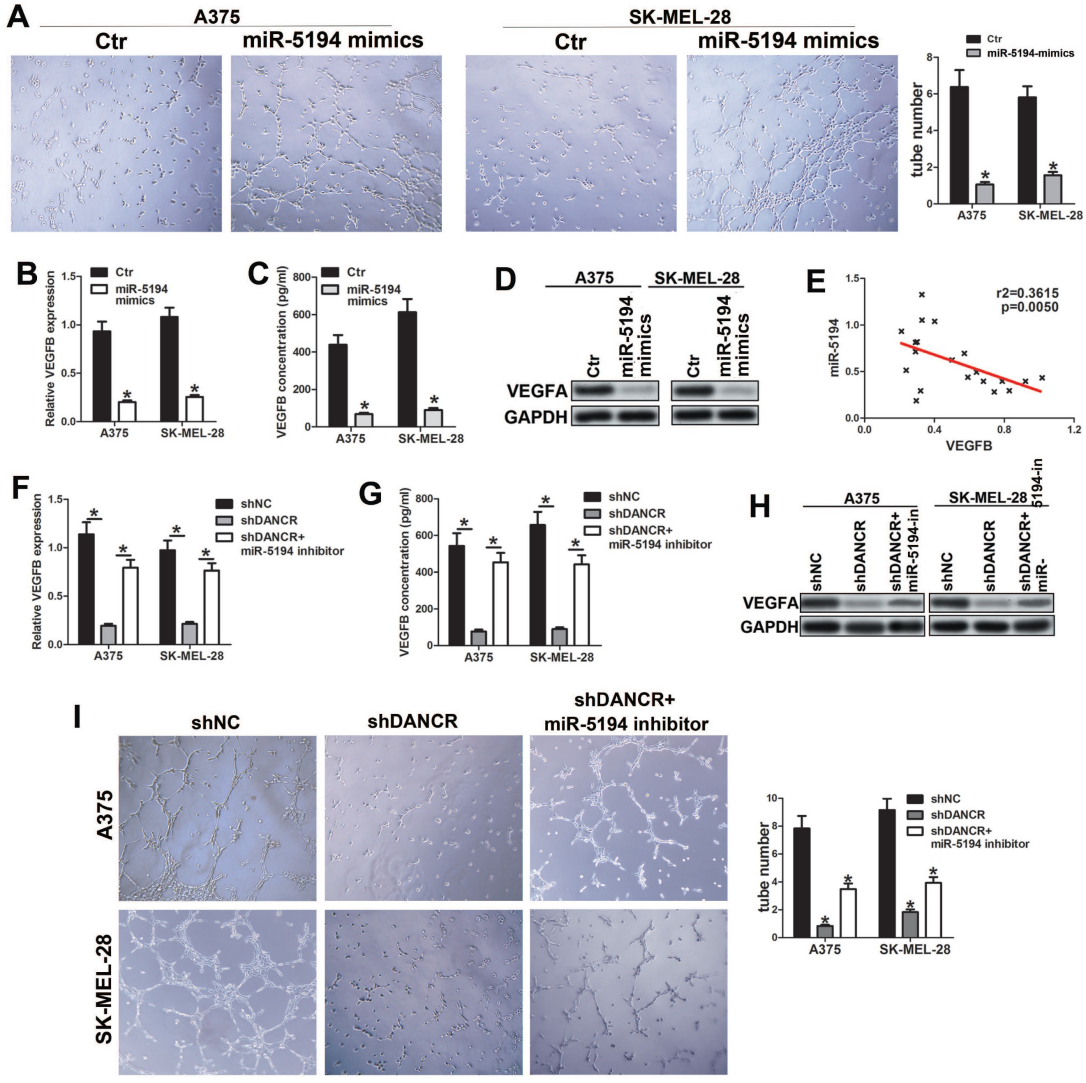
** miR-5194 plays crucial role in DANCR regulating VEGFB expression and angiogenesis. A.** Tube formation ability of HUVECs with treatment of CM from A375 and SK-MEL-28 cells with or without overexpression of miR-5194; Right panel: quantitative analysis of tube number. **B.** Concentration of secreted VEGFB in miR-5194 overexpressed and control A375 and SK-MEL-28 cells. **C** and **D.** Expression of VEGFB in above melanoma cells in mRNA level and protein level. **E.** Correlation of VEGFB and miR-5194 in human melanoma tissues. **F.** A375 and SK-MEL-28 cells were transfected with shDANCR separately or corporately with miR-5194 inhibitor (miR-5194-in). Secreted VEGFB was determined with ELISA assay. **G** and **H.** mRNA level and protein level of VEGFB were analyzed in above melanoma cells. **I.** Tube formation ability of HUVECs with treatment of CM from A375 and SK-MEL-28 cells with treatment of DANCR knockdown presence or absence of miR-5194 inhibitor; Right panel: quantitative analysis of tube number. *p < 0.05.

To investigate whether miR-5194 involved in DANCR regulating VEGFB, we overexpressed miR-5194 in melanoma cell (SFigure 3A) and found attenuated ability of promoting HUVECs tube formation in miR-5194-overexpressed (miR-5194-mimics) melanoma cells (Figure 7A). Furtherance, miR-5194 overexpression downregulated VEGFB secretion and expression in A375 and SK-MEL-28 cells (Figure 7B, 7C and 7D). Negatively correlation between VEGFB and miR-5194 expression further verified that miR-5194 could suppress VEGFB expression (Figure 7E). Next, we used miR-5194 inhibitor to treat DANCR knockdown melanoma cells and found that miR-5194 inhibitor successfully reversed the reduction of VEGFB secretion and expression induced by DANCR downregulation (Figure 7F, 7G and 7H). Besides, miR-5194 inhibitor lessened the suppression of HUVECs tube formation induced by DANCR knockdown (Figure 7I). Taken together, results in figure 7 confirmed the crucial role of miR-5194 in DANCR promoting VEGFB expression and neovascularization.

## Discussion

In this study, we analyzed the microarray data profiling from TCGA database and verified the tumor promoting role of lncRNA DANCR in melanoma progression. Further exploration revealed that except attenuating cell proliferation, downregulated DANCR also diminished angiogenesis of melanoma, which is an important way for supporting tumors with nutrients and oxygen. We further found that VEGFB played the key role in DANCR mediating angiogenesis. Mechanically, we confirmed that DANCR elevated VEGFB expression through directly binding and sponging miR-5194, inhibiting which could rescue DANCR knockdown inducing VEGFB suppression. In summary, our results suggested that DANCR facilitated VEGFB expression and the sequential angiogenesis to enhance melanoma progression through directly binding with miR-5194. Clarifying the function of DANCR in melanoma development may open a new avenue for melanoma therapy and broadened the understanding of DANCR in promoting cancer progression.

Angiogenesis, complex but highly controlled, is a process of blood vessels formation from the pre-existing vascular, which plays pivotal roles in tumor survival, development and metastasis 21. Therefore, angiogenesis is a critical indicator of poor clinical prognosis and tumor aggressiveness 22,23. In malignant tumors, genetic alternation is not sufficient for tumor progression. Acquisition of the ability to induce angiogenesis is necessary for neoplastic cells to satisfy their demands for oxygen and nutrients. The important role of angiogenesis in melanoma has been recognized, but the molecular mechanism of melanoma cell facilitating angiogenesis needs further clarification due to its character of complexity. In our study, we found dysregulation of DANCR played an oncogenic role in melanoma progression. Although DANCR knockdown suppressed cell proliferation *in vitro*, more significant efficiency of tumor suppression was found in the xenograft model. The pale appearance of xenograft indicated the angiogenic role of DANCR, which also contributed a lot in DANCR promoting melanoma progression.

Angiogenesis usually starts from sprouting from the existing vessels that keep quiescent without stimulation of pro-angiogenic factors, among which VEGFB is one of the most important one 20,24. Previous study also demonstrated the angiogenic role of DANCR in promoting several cancers progression 25,26. Establishment of abundant vascular network in the microenvironment is important in melanoma progression, especially in the phase of rapid growth. Thus, drugs inhibiting angiogenesis have been developed, such as bevacizumab. Unfortunately, usage of these anti-angiogenesis drugs failed to show effectiveness as initially expected due to the occurrence of resistance 27,28. One of the underlying mechanisms for the inefficiency is the highly overlapping character of pro-angiogenesis network; and the presence of various stimulatory factors and the downstream pathways and effectors. Hence, further clarification of molecular mechanisms responsible for angiogenesis of melanoma is needed. Our study revealed that DANCR increased angiogenesis in melanoma microenvironment through miR-5194/VEGFB signaling, targeting which might improve therapeutic effect.

Preliminary studies showed that miR-5194 could work as a tumor suppressor in Glioblastoma 29. Moreover, miR-5194 was an interim key step of circPARD3 increasing ENO1 in enhancing squamous cell carcinoma 30. Additionally, in melanoma cells miR-5194 could be sponged and repressed by LINC01063, through which SOX12 was upregulated 31. Except for cancer, during hepatic lipid metabolism, miR-5194 distributing in the mitochondria activated ADIPOR1/PPARA signaling that sequentially accelerated fatty acid oxidation 32. In present study, we demonstrated that miR-5194 overexpression reduced VEGFB expression and DANCR directly bound and sponged with miR-5194 to upregulate VEGFB. Although the function of miR-5194 in melanoma has been partly reported previously 31, our study further complemented its role in modulating angiogenesis as well as its tumor-suppression role in melanoma progression.

In conclusion, our study revealed the promotional role DANCR played in melanoma progression and neovascularization in melanoma microenvironment. Mechanically, DANCR directly interacted with miR-5194 and sponged it to diminish the suppression of miR-5194 on VEGFB expression. Therefore, DANCR might be a novel target for improving melanoma treatment.

## Supplementary Material

Supplementary figures and tables.

## Figures and Tables

**Figure 8:**
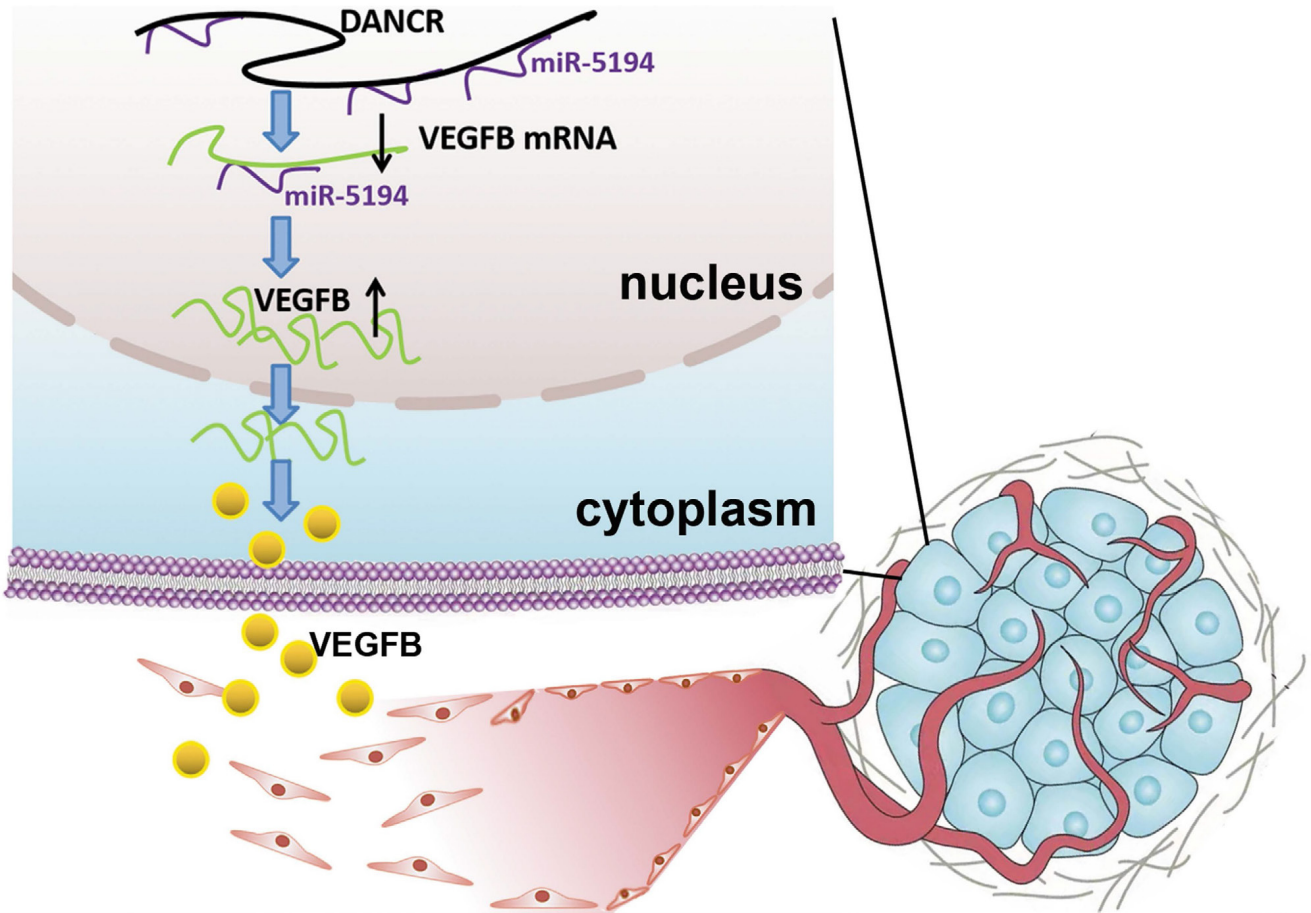
** Schematic representation of the mechanism underlying DANCR enhancing angiogenesis in melanoma.** DANCR sponged miR-5194 to upregulated VEGFB, which sequentially enhanced angiogenesis in melanoma.

**Table 1:** Clinicopathologic characteristics of patients

Characteristic	High DANCR expression	Low DANCR expression	Total (n=20)	*p*-Value *
	n (%)	n(%)		
Age				0.659
≤40 years	5(62.50%)	3(37.50%)	8 (40%)	
>40 years	5(41.67%)	7(58.33%)	12 (60%)	
Gender				0.572
Male	7(63.63%)	4(36.36%)	11 (55%)	
Female	3(33.33%)	6(66.67%)	9 (45%)	
Tumor stage				0.046*
pTis, pT1, pT2	1(12.50%)	7(87.50%)	8 (40%)	
pT3-pT4	9(75.00%)	3(25.00%)	12 (60%)	
M-stage				0.079
M0	6(40.00%)	9 (60.00%)	15	
M1	4(80.00%)	1(20.00%)	5	
Lymph node involvement				0.692
N0	2(40.00%)	3(60.00%)	5	
N1	4(57.14%)	3(42.86%)	7	
N2	4(50.00%)	4(50.00%)	8	
Clark level value				0.493
I+II	1(33.33%)	2(66.67%)	3	
III	2(33.33%)	4(66.67%)	6	
IV	3(42.86%)	4(57.14%)	7	
V	4(100%)	0(0%)	4	

## Abbreviations

Lnc RNA: Long non-coding ribonucleic acid
DANCR: Differentiation antagonizing non-protein coding RNA
ceRNA: competing endogenous ribonucleic acid
siRNA: small interfering ribonucleic acid
miRNA: micro ribonucleic acid
shRNA: short hair ribonucleic acid
mRNA: massage ribonucleic acid
EZH2: Enhancer Of Zeste Homolog 2
TIMP: Tissue inhibitor of matrix metalloproteinase
SFM: serum free medium
CM: conditioned medium
PBS: phosphate buffered saline
IHC assay: immunohistochemical assay
TCGA: the cancer genome atlas
VEGF: vascular endothelial growth factor
qRT-PCR: Real-time quantitative-polymerase chain reaction
MTT: methyl thiazoly tetrazolium
ELISA: enzyme-linked immuno sorbent assay
HUVEC: Human Umbilical Vein Endothelial Cells
DANCR-mt: mutant-DANCR
