# Supplementary material for: LncRNA DANCR Enhances Angiogenesis to Promote Melanoma Progression Via Sponging miR-5194
Source: J Cancer. 2023 May 5;14(7):1161–73. doi: 10.7150/jca.81723 (PMC10197948; doi:10.7150/jca.81723)
Supplement: Supplementary file 1 — Supplementary figures and tables. [file jcav14p1161s1.pdf]

Supplementary Material

Supplementary Figures

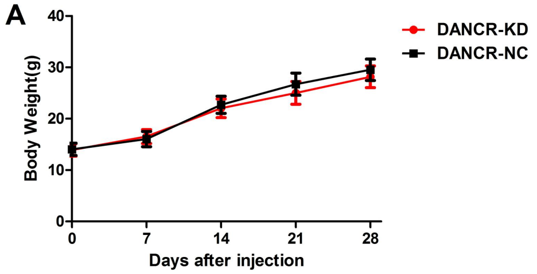

Supplementary Figure 1. Body weight of the mice carrying subcutaneous tumors constituted by different cells.

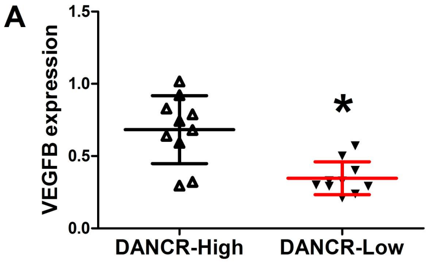

Supplementary Figure 2. Quantitative analysis of VEGFB expression in melanoma tissues with lower and higher DANCR level. *p<0.05.

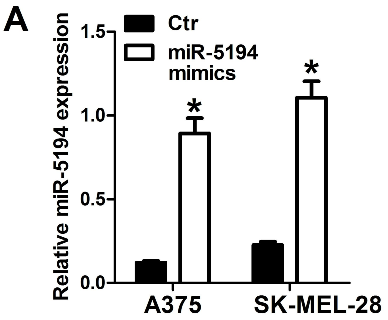

Supplementary Figure 3. Expression of miR5194 in A375 and SK-MEL-28 cells with transfection of miR5194-mimics and miR-Ctr (Ctr).
